# Hi-C guided genome assembly and karyotype analysis of the Indian population of *Maruca vitrata* (Lepidoptera: Crambidae) set the stage for structural and functional genomic insights

**DOI:** 10.1186/s12863-026-01446-2

**Published:** 2026-06-13

**Authors:** Muthugounder Mohan, P. J. Aneesha, Karuppannasamy Ashok, R. Gandhi Gracy, T. Venkatesan, Sagar Doddchowdappa, Kesavan Subaharan, Suresh Ramakrishana Jambagi, B. Anilkumar, N. Vijayakumari, C. R. Chandana, S. N. Sushil, Ramasamy Srinivasan

**Affiliations:** 1 Division of Genomic Resources, ICAR- National Bureau of Agricultural Insect Resources, Bengaluru, 560 024 India; 2 Division of Entomology, ICAR–Indian Agricultural Research Institute, New Delhi, 110012 India; 3 World Vegetable Center, Shanhua, Tainan, 74199 Taiwan

**Keywords:** *Maruca vitrata*, Chromosome-level genome, Gene prediction, Annotation

## Abstract

**Objectives:**

The spotted or legume pod borer, *Maruca vitrata* (Lepidoptera: Crambidae), is a global insect pest of many economically important grain and vegetable legume crops. Although the species is believed to have originated in the Indo-Malayan region, populations in India have shown evidence of genetic divergence, potentially representing distinct cryptic species or host-associated races. A comprehensive understanding of the evolutionary relationships and functional divergence within *M. vitrata* is currently limited by the absence of detailed genomic resources. Integrating karyotype data with high-resolution genomic information is therefore essential to elucidate the genetic architecture and evolutionary dynamics of the Indian lineage of *M. vitrata* and to inform effective pest management strategies.

**Data description:**

PacBio HiFi sequencing and proximity ligation technique (Hi-C), we provide here an improved genome assembly of *M. vitrata*. The 459.3 Mb genome was assembled into 45 scaffolds with a scaffold N50 of 15.6 Mb. The 31 pseudochromosomes (size range from 9.48 to 20.42 Mb) accounted for 458 Mb, representing 99.73% of the genome assembly. The genome has a repeat content of 36.67% and 21,586 protein-coding genes. The genome completeness analysis using BUSCO with the Insecta lineage((Insecta_odb10) captured 99.5% of the complete BUSCOs. The karyotype analysis revealed the presence of 31 chromosomes (1n). This genome-scale data helps by identifying target genes for the development pest management strategies in the future. The 31 pseudochromosomes correspond to the 31 chromosomes identified through karyotype analysis, thereby confirming the chromosomal-level completeness and structural accuracy of the assembly.

## Objective

The legume pod borer, *Maruca vitrata* F. (Lepidoptera: Crambidae), is the most devastating insect pest of many food legumes, and it is one of the most widely distributed insect species in the world [1]. Its larvae feed on 73 wild and cultivated legume host plant species of the family Fabaceae [2]. Female adult moths lay eggs on flowers, buds, leaves, and twigs. Early instar larvae prefer to feed on flowers and flower buds, whereas later instar larvae make webs on flowers, buds, blossoms, and pods with adjacent leaves and bore into developing buds and pods. In the field, *M. vitrata* occurs with overlapping generations if host plants are continuously present, leading to complete crop failure when no protection measures are taken [3]. A yield loss of up to 72%- 84% was observed in cowpea and pigeon pea [4, 5]. In Taiwan, it has been reported yield losses range from 17% to 53% for cowpea [6] and 10% to 45% for mungbean in Bangladesh [7]; 25% to 40% for yard-long bean in Cambodia, Indonesia, and Thailand [8–10]. Many cryptic species within *M. vitrata* are suspected due to differential responses in male moths to same sex pheromone blends [11–14]. The nuclear ribosomal internal transcribed spacer 2 (ITS2) marker conveniently differentiated *M. vitrata* populations occurring in Asia and Africa [15]. Phylogenetic analysis of *Maruca* populations from different continents using the COX1 marker derived from mitochondrial cytochrome c oxidase-I also confirmed the presence of subspecies among the *M. vitrata* populations existing in different continents [16, 17]. Later, the occurrence of two putative subspecies (Asia and Africa) that responded differently to the sex pheromone was predicted using pheromone-binding protein (PBP) genes [18].

These molecular investigations have revealed significant genetic diversity among *M. vitrata* populations from different continents, with indications of regional structuring and restricted gene flow. Notably, the Indian population has exhibited unique molecular signatures in some studies, suggesting a possible divergence from its African and Southeast Asian counterparts. However, comprehensive cytogenetic comparisons remain limited. A recent study on comparative genomic analysis on chromosome evolution revealed that more than 80 per cent of lepidopteran species have 28–31 chromosomal numbers (1n), which are ancestral, whereas the remaining species have undergone extensive reorganisation [19]. Identification of such structural genomic differences will not only help in understanding the evolutionary history of *M. vitrata* but also has practical implications for its management, especially in the context of region-specific control strategies. This study presents a high-resolution, chromosome-scale genome assembly of *Maruca vitrata* from the Indian subcontinent, generated using highly accurate PacBio HiFi long reads and scaffolded with Hi-C data. Integrated with cytogenetic karyotyping, these findings aim to clarify whether the Indian lineage of *M. vitrata* represents a cytogenetically.

## Data description

The pre-starved early fourth instar larvae from an iso-female colony of *M. vitrata* (National Accession No: NBAIR-IS-CRA-02), maintained at ICAR-National Bureau of Agricultural Insect Resources (NBAIR), Bengaluru, India, for over 100 generations, were used for isolation of genomic DNA.For long-read sequencing, high-molecular-weight (HMW) genomic DNA was extracted from the antenna of female tissue using the Monarch HMW DNA Extraction Kit (New England Biolabs, MA, USA), following the manufacturer’s instructions. The HMW genomic DNA was used for genome sequencing to create a library with an approximate insert size of 20 Kb for the PacBio Revio System utilizing SMRT Bell Express Template Prep Kit 2.0, following the manufacturer’s recommendations. The library was sequenced using a single SMRT Cell on the PacBio Revio System (Pacific Biosciences, CA, USA)(Data file 1). For short-reads Illumina whole-genome sequencing, paired-end libraries with insert sizes of 250 bp were prepared and sequenced on Illumina Novaseq 6000 platform following the standard Illumina protocols(Data file 2).

The Hi-C sequencing library was generated using Arima kit following the manufacturer’s instructions. This method involved cross-linking ground female *M. vitrata* samples, isolating nuclei, and digesting with DpnII and HinfI. DNA fragments were biotin-labeled, sheared to 300 ~ 600 bp, blunt-end repaired, A-tailed, and then selected via biotin-streptavidin pull-down and after 12 ~ 14 cycles of PCR amplification, the library was sequenced on the Novaseq 6000 platform(Data file 3). In addition Long read transcriptome sequencing was done using Pacbio platform. RNA was isolated from the adult, larva, and pupae of *M. vitrata* using an RNAeasy kit (Qiagen) as per the manufacturer’s instructions. For PacBio Iso-Seq RNA with > 7 RIN was taken and according to the recommendations provided by the manufacturer, a full-length cDNA library was constructed using SMRTbell prep kit 3.0 from RNA and then the full-length cDNA was amplified by PCR. Its damage and ends were repaired and sequenced on the PacBio Revio System (Pacific Biosciences, CA, USA)after qualification(Data file 4).

The genome survey using K-mer analysis [20] indicated the genome size as 482 Mb with a heterozygosity value of 5.26%(Data file 5). After checking the quality of raw reads, 96.7 and 80.9 GB of filtered PacBio and Illumina data, with5,362,460 and 42,548,514 clean reads were generated. The Hifiasm assembler (v0.19.5) [21] assembled into a 540 Mb primary-level contig assembly with an N50 value of 14.0 Mb. The Purge_Dup tool v1.2.6 [22] was employed to remove the duplicates, which led to a reduction of total assembly size of 465 Mb. Further, the NCBI-FCS tool [23] was run to remove contaminants. To construct a chromosome-scale reference assembly, additional scaffold refinement was performed using approximately 100 million paired‐end reads from the Hi‐C sequencing.

The integration of Hi-C data using the tools HiCUPv0.92 [24] and YAHS [25] enabled the resolution of the genome into larger, pseudochromosomes. This integration generated 45 scaffolds totalling 459.3 Mb of genome with an N50 of 15.6 Mb. The 31 pseudochromosomes, which are greater than 9.0 Mb, contribute to 458.3 Mb of the genome, reflecting a chromosomal-scale assembly representing 99.73% of the assembled genome (Data file 6,7). The final genome assembly was subjected to the Benchmark of Universal Single-Copy Orthologs (BUSCO) assessmentv5.4.7 [26] for the estimation of genome completeness revealed 99.6% completeness under Insecta _db10 database (Orthodb v10) (Data file 8). This also included 99% complete and single-copy BUSCO genes and 0.5% complete and duplicated BUSCO genes.

Telomeric regions within the *Maruca vitrata* genome were identified using the Telomere Identification Toolkit [Tidk (v0.2.0); https://github.com/tolkit/telomeric-identifier*]* [27]. Telomeric repeat sequences were identified at both ends of the *Maruca vitrata* genome in 18 chromosomes. Twelve chromosomes showed telomere signals at only one end, whereas the remaining single chromosome did not show any signatures of telomeric presence, suggestive of degeneration (Data file 9).

The mitotically active imaginal discs were dissected from the 4th instar larvae of *M. vitrata.* Cells were treated with a 0.025% colchicine solution to arrest them in metaphase, followed by 1% sodium citrate hypotonic treatment to swell the cells and spread the chromosomes as per Imai et al., 1988 [28]. After fixationusing cornoy’s fixative (3:1- methanol: glacial acetic acid), cell suspensions were dropped onto microscope slides and stained using aceto-carmine and giemsa staining. More than 20 Metaphase spreads were examined under a light microscope for counting the number of chromosomes and 10 were counted as 31 chromosmes under 1000x magnification in 100X oil immersion. (Data file 10) (Table 1).

**Table 1:** Overview of data files/data sets

Label	Name of data file/data set	File types (file extension)	Data repository and identifier (DOI or accession number)
Data file 1	Raw long whole genome Hi-Fi	Fasta file (.fastq)	http://identifiers.org/insdc.sra:SRX25648789 [33]
Data file 2	Raw short whole-genome Illumina sequencing reads	Fasta file (.fastq)	http://identifiers.org/insdc.sra:SRX25648790 [34]
Data file 3	Raw Hi-C reads	Fasta file (.fastq)	http://identifiers.org/insdc.sra:SRX32328014 [35]
Data file 4	RawLong RNA-seq reads	Fasta file (.fastq)	http://identifiers.org/insdc.sra:SRX33071967 [36]
Data file 5	K- mer plot of short reads File name: Kmer-Mar-SR	Image file (.png)	Figshare, 10.6084/m9.figshare.29908724 [37]
Data file 6	Assembled genome	Fasta file (.fa)	http://identifiers.org/insdc.gca:GCA_041475385.2 [38], https://identifiers.org/ncbi/insdc:JBFTXK020000000[39]
Data file 7	Hi-C clustering heat map File name: Hi-C interaction	Image file (.jpeg)	Figshare, 10.6084/m9.figshare.29908724 [37]
Data file 8	BUSCO assessment of the assembly using the Insecta database File name: Busco_insecta_odb10	7z file (.7z)	Figshare, 10.6084/m9.figshare.29908724 [37]
Data file 9	Telomere Identification File name: Mar-tidk-plot; telomere identification	svg files (.svg)	Figshare, 10.6084/m9.figshare.29908724 [37]
Data file 10	100X karyotyping image of *Maruca vitrata* brain cells using Aceto carmine staining using the squash and break method. File name: karyotyping images; karyotyping MV	PDF, Zip files (.pdf, .zip)	Figshare, 10.6084/m9.figshare.29908724 [37]
Data file 11	Repeat region File name: Predicted repetitive sequences	Gff file (.gff)	Figshare, 10.6084/m9.figshare.29908724 [37]
Data file 12	Coding gene sequence File name: Predicted gene - CDS	Fasta file (.fas)	Figshare, 10.6084/m9.figshare.29908724 [37]
Data file 13	Protein sequence File name: Predicted gene - Protein	Fasta file (.fas)	Figshare, 10.6084/m9.figshare.29908724 [37]
Data file 14	gene locations File name: Predicted gene	Gff3 file (.gff3)	Figshare, 10.6084/m9.figshare.29908724 [37]
Data file 15	Genome feature statistics File name: N50; Size	txt files (.txt)	Figshare, 10.6084/m9.figshare.29908724 [37]
Data file 16	Gene annotation using GO, KO, InterPro, Pfam, KEGG, SwissProt, Meryl, and SwissProt databases File name: annotation	text file (.txt)	Figshare, 10.6084/m9.figshare.29908724 [37]

Earl Grey v6.3 at default parameters [29] has been used to identify repeats, which comprise 168,023,698 bp (36.58%) and were masked in the assembled genome of 459.3 Mb(Data file 11). The repeats comprise unclassified elements accounting for 11.8% of the whole genome, followed by long terminal repeats of 11.03%. Structural gene prediction was performed using the BRAKER3 (v3.0.8) pipeline [30] on the soft-masked draft genome. This pipeline integrates both ab initio and homology-based approaches for gene prediction. Protein sequences from Crambidae family were retrieved from the RefSeq protein databases to provide additional support for gene prediction(Data file 12–15). Additionally, ISO-SEQ RNA-seq data generated from pooled samples which was mix of Larvae, Pupae and adults were also used as evidence for gene prediction. The genome comprises 21,586 genes derived from 48,959 transcripts, as evidenced by RNA-seq data, which utilizes filters greater than 300 bp. The functional annotation using InterProScanv5.72-103.0 [31] and homologous sequences were identified using the blastp (v2.16.0+) [32] program with an e-value cutoff of 10^− 5^, ensuring statistically significant matches, revealed 11,230 and 17,615 annotated genes, respectively(Data file 16).

The genome sequencing data, combined with cytogenetic karyotyping, revealed 31 pseudochromosomes corresponding to the 31 chromosomes observed in the Indian lineage of *Maruca vitrata*. These results indicate that the Indian *M. vitrata* population is both cytogenetically and genomically distinct from populations in other geographic regions.

Although we generated a chromosome-level assembly, there remains scope for further improvement. Several gaps are still present in the assembly, and telomeric signals were not detected in some pseudochromosomes; in others, telomeric repeats were identified on only one chromosomal arm. This may partly reflect limitations of the tool TIDK, which is not species-specific but rather designed at the family level. In addition, centromeric regions could not be resolved in the current assembly. Therefore, further refinement of the genome assembly is possible. Such improvements could be achieved using ultra-long read sequencing technologies, and genome size estimation could be independently validated using flow cytometry.

### Limitations

This Data Note presents the whole-genome sequencing of *Maruca vitrata*, based on a combination of the PacBio HiFi and Illumina NovaSeq 6000 platforms, providing a high-quality, improved genome assembly over the previous version. Therefore, the authors believe that there are no limitations in the data.

## Data Availability

Sequence Read Archives were deposited in NCBI with accession number SRP525227 ; whole genome shotgun sequencing project was submitted in GenBank (JBFTXK020000000), and genome assembly at NCBI genome (GCA_041475385.2). Other data related this genome was uploaded in figshare (https://doi.org/10.6084/m9.figshare.29908724).

## Abbreviations

*M. vitrata*: *Maruca vitrata*
